# Stewardship of personal protective equipment (PPE): An important pandemic resource for PPE preservation and education

**DOI:** 10.1017/ice.2020.311

**Published:** 2020-06-24

**Authors:** Ami B. Patel, Anna O’Donnell, Amanda Bonebrake, Matthew McHugh, Katrina Espiritu, Molly Steele, Maria Bovee, Robert Jones, Karen Richey, Genevieve Frey, Kathleen English, Jade Tucker, Larry K. Kociolek

**Affiliations:** 1 Department of Pediatrics, Ann & Robert H. Lurie Children’s Hospital of Chicago, Chicago, Illinois; 2 Northwestern University Feinberg School of Medicine, Chicago, Illinois

*To the Editor—*Healthcare workers (HCWs) are at high risk of COVID-19 because of prolonged close contact with SARS-CoV-2–infected patients. Personal protective equipment (PPE), particularly respirators for care of patients undergoing aerosol-generating procedures (AGPs), is fundamental in protecting HCWs. However, global PPE shortages have occurred during this crisis, forcing healthcare institutions to consider alternative PPE management approaches with regard to appropriate utilization and conservation. At our institution, we limited PPE distribution from central supply to avoid misuse and to maintain visibility on usage and needs. This approach led to delays in obtaining PPE by bedside teams. Furthermore, frequent changes in public health guidance and hospital PPE approaches resulted in HCW confusion and apprehension. To address these issues, we implemented several strategies to optimize PPE utilization and education. The most valuable was a PPE stewardship initiative, which allowed us to simultaneously reserve PPE, assure HCW safety, provide real-time education and guidance regarding optimal infection control practices and PPE use, and ensure timely PPE availability. Here we present our successful PPE stewardship initiative at the Ann & Robert H. Lurie Children’s Hospital of Chicago.

Early in the pandemic, PPE stewardship was the responsibility of the infection prevention and control (IP&C) team, who instated daily bedside COVID-19 rounds on all suspected and confirmed COVID-19 inpatients. IP&C provided patient-specific recommendations regarding isolation status and PPE utilization, the recommendations for which frequently changed based on new science and evolving public health guidance. Rounds were conducted as a multidisciplinary team that included IP&C, respiratory therapists, bedside and quality nurses, physicians, and unit leadership. These rounds allowed for discussion and dissemination of current PPE guidance and fostered discussions regarding risk mitigation practices. The benefits were reciprocal—clinicians provided patient-related information to clarify the level of PPE required, and in return, IP&C provided PPE feedback and education regarding PPE needed, how to don and doff, handling N95s for reuse/extended use, and identifying additional PPE preservation opportunities. Decisions regarding PPE use, isolation status, and room type (ie, need for an airborne infection isolation room) were made with consideration of anticipated AGPs and respiratory interventions. We differentiated high-risk and low-risk AGPs^1,2^ to strategically use respirators for the patients at highest risk (Table 1). Respirators were recommended if a high-risk AGP was anticipated in the next 24 hours. To further preserve PPE, a patient-specific action plan was identified to expedite stepping down isolation when COVID-19 tests results were negative.

**Table 1.** List of Procedures Based on Risk for Aerosol Generation

Risk	Procedure
Low-risk AGP	Nasopharyngeal/oropharyngeal sample collection Metered-dose inhaler Insertion of nasogastric/orogastric tube Closed or nasal/oropharyngeal suctioning
High-risk^a^ AGP	Open airway deep or tracheal suctioning (below vocal cords) Mechanical ventilation Tracheal intubation or extubation Laryngeal mask airway Noninvasive ventilation (BiPAP, CPAP, HFNC) Tracheostomy placement/patient with tracheostomy Cardiopulmonary resuscitation Bronchoscopy Sputum induction/cough assist Nebulizer therapy Cool mist humidification

Note. BiPAP, bilevel positive airway pressure; CPAP, continuous positive airway pressure; HFNC, high-flow nasal cannula.

a N95/CAPR recommended only for high-risk aerosol-generating procedures.

As the pandemic evolved in Chicago, the volume of suspected and confirmed COVID-19 cases increased, and the need to extend our PPE stewardship initiative, particularly to overnight teams, became evident. Thus, nursing leadership launched a team to extend and further support IP&C and PPE stewardship efforts. This new team, the “PPE Spotters,” was composed of bedside and quality nurses and respiratory therapists with the following goals: (1) to work closely with supply chain team to monitor and optimize PPE utilization, supply, and distribution; (2) to provide “just-in-time” training related to PPE donning/doffing and PPE reuse; and (3) to provide general support to hospital staff for COVID-19–related questions. The team was available 24/7 through a dedicated mobile phone to provide resources such as PPE, testing supplies, and educational videos and handouts. They performed bedside rounds with the care teams for suspected and confirmed COVID-19 inpatients to answer PPE-related questions, to deliver and evaluate bedside PPE supply, to collect unused PPE for return to central supply, and to ensure PPE utilization aligned with patient-specific care plans. The PPE Spotters had a profound impact on supply chain management by consolidating demand for the entire hospital to a single stocking location, the PPE Spotter cart. This approach allowed supply chain and central supply to anticipate demand and avoid periodic and unpredictable depletion of critical supplies such as respirators and respiratory viral specimen collections kits. In addition to their partnership with inpatient caregivers, the PPE spotters provided support to the emergency department, the transport team, anesthesia services, and procedural services.

We measured the number of patient days that confirmed or suspected patients with COVID-19 remained under isolation, in addition to numbers of N95 masks distributed to hospital units from central supply during the 4-week period before and the 8-week period after the PPE Spotter team was formed. Before the PPE Spotter team was implemented, the central supply service distributed 18.2 masks per COVID-19 isolation patient-day (ie, 2,345 masks during 129 COVID-19 isolation patient days over 4 weeks) and after the PPE Spotter team was implemented, the central supply service distributed 3.0 masks per COVID-19 isolation patient-day (ie, 3,955 masks during 1,300 COVID-19 isolation patient days over 8 weeks). Thus, our PPE stewardship efforts successfully limited N95 misuse as community COVID-19 burden increased, despite rising inpatient volumes of confirmed COVID-19 and enacting universal COVID-19 admission testing, which required ~8–24 hours of COVID-19 isolation for all inpatients pending test results.

Following the intervention, IP&C met with hospital units and departments to discuss successes and areas for improvement in our ongoing hospital COVID-19 response. A consistent theme was that the PPE Spotter team was very well received and fulfilled the intended educational and PPE preservation goals. Stakeholders identified IP&C and the PPE Spotter team as an excellent multidisciplinary resource, particularly during periods of evolving PPE guidance and supply chain insecurity. An additional benefit of this team was further expanding the pool of hospital staff to advocate for HCW safety and IP&C principles. We are working to determine how we can continue a similar model as COVID-19 activity decreases to maintain a readily available resource to bedside healthcare providers. In summary, our stewardship initiatives for PPE were successful in minimizing PPE misuse, and they provided a conduit for real-time education to frontline providers.
